# Multifaceted Role of PARP-1 in DNA Repair and Inflammation: Pathological and Therapeutic Implications in Cancer and Non-Cancer Diseases

**DOI:** 10.3390/cells9010041

**Published:** 2019-12-22

**Authors:** Simonetta Pazzaglia, Claudio Pioli

**Affiliations:** ENEA, Laboratory of Biomedical Technologies, 00123 Rome, Italy

**Keywords:** PARP, inflammation, tumor, DNA repair, PARP inhibitors

## Abstract

PARP-1 (poly(ADP-ribose)-polymerase 1), mainly known for its protective role in DNA repair, also regulates inflammatory processes. Notably, defects in DNA repair and chronic inflammation may both predispose to cancer development. On the other hand, inhibition of DNA repair and inflammatory responses can be beneficial in cancer therapy and PARP inhibitors are currently used for their lethal effects on tumor cells. Furthermore, excess of PARP-1 activity has been associated with many tumors and inflammation-related clinical conditions, including asthma, sepsis, arthritis, atherosclerosis, and neurodegenerative diseases, to name a few. Activation and inhibition of PARP represent, therefore, a double-edged sword that can be exploited for therapeutic purposes. In our review, we will discuss recent findings highlighting the composite multifaceted role of PARP-1 in cancer and inflammation-related diseases.

## 1. Introduction

Poly(ADP-ribose)-polymerases (PARPs), more recently named ADP-ribosyl-transferase diphtheria toxin-like (ARTDs), belong to an ancient evolutionary-conserved family of proteins involved in abiotic and biotic stress responses, DNA repair, cell death, division, and differentiation, as well as in inflammation and immune responses. Seventeen PARPs have been described, all of which utilize nicotinamide (NAD^+^) as substrate but have distinct structural domains, activities, subcellular localizations, and functions [1,2,3,4].

PARP-1, the prototypical member of the family, is characterized by a zinc-finger DNA binding domain, an auto-modification domain and a catalytic domain, which adds branched ADP-ribose moieties to target proteins [5]. By mean of poly-ADP-ribosylation (PARylation), PARP-1 negatively regulates its own enzymatic activity [6] and modifies functions of acceptor proteins due to the high negative charge and complexity of ADP-ribose polymers (PARs) [3]. Acceptor proteins include PARPs, histones, DNA repair enzymes, chromatin regulators, and transcription factors. PARP-1 regulates target proteins not only through enzymatic modifications but also by acting as a docking molecule. This also occurs for transcription factors as it is the case of NF-κB [7], a transcription factor family linking PARP-1, DNA repair, and inflammation.

Upon binding to DNA lesions, PARP-1 becomes catalytically active, thus acting as a DNA damage sensor. PARP-1 is responsible for 80–90% of the global PAR synthesis following DNA strand breakage [8]. The structural analysis of PARP-1 has provided insights into how PARPs recognize DNA damage and how DNA damage detection allosterically leads to robust production of poly(ADP-ribose) [9,10,11]. Many PARP inhibitors (PARPis) bind PARP-1 in an orientation resembling that of its substrate NAD^+^, and lock the catalytic site, preventing autoPARylation, and thereby, trapping PARP-1 in the damage site [12].

PARP-1 regulates gene expression under basal as well as signal- and stress-activated conditions, at multiple levels: chromatin structure, methylation pattern, enhancer-binding, promoter co-regulation, activation of transcription factors, insulation, and post-transcriptional RNA modifications [13,14,15]. PARP-1 (as other family members) is also activated in response to signals independently from DNA damage. The extracellular signal-activated kinase (Erk) phosphorylates PARP-1 modulating its activity and consequently affecting NF-κB PARylation levels [16]. Although autoPARylation is the most important modification, PARP-1 is also regulated by other post-translational modifications. PARP-1 enzymatic activity and its ability to interact with DNA, transcription factors and other proteins are regulated by phosphorylation, methylation, acetylation, sumoylation, and ubiquitination [16,17,18].

PARs are fast removed and reduced to monomers by PAR hydrolase (ARH) and poly(ADP-ribose)-glycohydrolase (PARG), establishing a dynamic equilibrium in the PARylation status of targeted proteins. As PARs (either free or protein bound) are recognized by specific motifs present on other proteins, their synthesis by PARPs and degradation by ARH/PARG also allow PARs to act as signal transducers. PARP-1, therefore, represents an important enzyme fast modulating responses according to environmental cues, under physiological as well as pathological conditions. 

We here intend to review the main roles of PARP-1 in DNA repair and inflammation, and the pathological involvement of this multifaceted molecule in cancer and inflammatory-related diseases. A brief account of PARPis counteracting the unwarranted activation of PARP-1 in cancer and non-cancer diseases and the consequent potential oncogenic risk of PARP inactivation is also discussed.

## 2. PARP-1 in DNA Repair

Cells are exposed to various endogenous and exogenous insults that induce DNA damage, which, if unrepaired, impairs genome integrity and leads to the development of various diseases, including cancer. PARP-1 is an abundant nuclear chromatin-associated protein endowed with a high DNA damage–sensing ability. Once encountering free DNA ends, PARP-1 is catalytically activated and generates large amounts of PARs which act as scaffolds for the recruitment of DNA repair enzymes or auto-modifies itself inducing dissociation from DNA damage site and localization of repair proteins to the lesion. Both generation of PAR following metabolic, oxidative, or genotoxic stresses, and PAR degradation are extremely rapid processes [19]. The prompt turnover of PAR is crucial for efficient DNA repair [20]. Defects in PAR catabolism result in increased DNA damage and are deleterious to cells [21]. 

**Figure 1:**
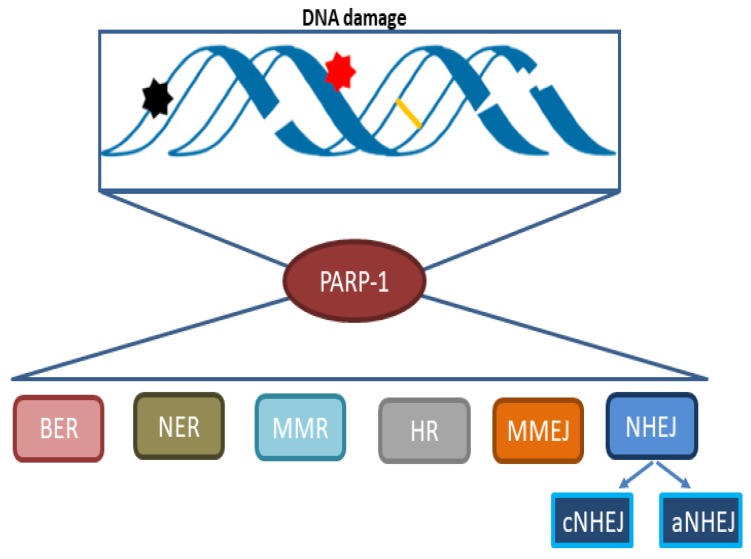
PARP-1 (poly(ADP-ribose)-polymerase 1) plays a role in multiple DNA damage recognition and repair pathways. BER, base excision repair; NER, nucleotide excision repair; MMR, mismatch repair; HR, homologous recombination; MMEJ, microhomology-mediated end-joining; NHEJ, non-homologous end-joining; cNHEJ, classical non-homologous end-joining; aNHEJ, alternative non-homologous end-joining.

PARP-1 was originally described as part of the base excision repair (BER) pathway based on genetic studies, but more recent results unveiled a wider role for PARP-1 and PARylation including all major DNA repair pathways (Figure 1). PARP-1 is involved in the regulation of nucleotide excision repair (NER) [22], classical non-homologous end-joining (cNHEJ) [23], alternative non-homologous end joining (aNHEJ) [24], microhomology-mediated end-joining (MMEJ) [25], homologous recombination (HR) [26], DNA mismatch repair (MMR) [27], and maintenance of replication fork stability [28]. The understanding of PARP-1 contribution to DNA damage repair continues to grow in more specific and mechanistic ways. It is now clear that PARP-1 regulates many cellular functions, which include multiple pathways of DNA repair [29]. Recently, it was also shown that PARP-1 recognizes the unligated Okazaki fragments and promotes repair during DNA replication [30]. As targeting the DNA damage response (DDR) machinery is an attractive strategy for designing novel chemotherapeutics, there has been an intense clinical focus on PARP-1 in the past few years. Mechanistically, defining PARP-1 dependent DNA processing functions is pivotal for the development of successful PARPi therapy and therapeutic regimens in the treatment of different cancers.

## 3. PARP-1 and Its Pro-Inflammatory Role

Inflammation is the first reaction of tissues in response to harmful stimuli, such as pathogens, damaged cells, or other stressors. Inflammation raises the alarm, inducing production of several factors, altering blood vessels permeability, recruiting leukocytes, and creating the context for activation of innate and (then) adaptive immune responses. Acute inflammation is a protective process normally resulting in removal of the initial damaging cause and dead cells, and ultimately leading to resolution and tissue healing. In contrast, when initial stressing factors are not (or cannot be) removed, the resolution phase of inflammation does not occur and unnecessary by-stander tissue damages further fuel inflammatory processes. This long-term, low level, chronic inflammation is associated with the onset and/or worsening of several diseases, including cancer, arthritis, colitis, diabetes, atherosclerosis, and neurodegenerative diseases. Noteworthy, PARP-1 knockout (KO) mice are protected by all these inflammatory/immune-mediated diseases as shown in several experimental models [2,31]. Chronic inflammation, either induced by environmental exposures, autoimmune/inflammatory diseases or chronic infections, is involved in both cancer development and progression as demonstrated by experimental and clinical studies [32,33]. Yet, the role of PARP-1 in cancer onset is more composite, due to its roles in inflammation and DNA damage recognition/repair (see below).

PARP-1 promotes inflammatory responses by positively regulating the pro-inflammatory NF-κB transcription factors. Oxidative stress, bacterial products (LPS) and inflammatory cytokines (IL-1, TNFα), all of which activate PARP-1, also activate NF-κB. PARP-1 sustains Toll-Like Receptors (TLRs)-induced NF-κB activation [34], a pathway involved not only in inflammation but also in carcinogenesis [35]. NF-κB activation and nuclear translocation require phosphorylation of I-κB inhibitors by the IκB kinase (IKK), an enzymatic complex including the regulatory element IKKγ, also known as NF-κB essential modulator (NEMO). PARP-1 is involved in DNA damage-induced sumoylation and consequent mono-ubiquitination of NEMO, which in turn triggers phosphorylation of I-κBα by IKK, and thus, NF-κB nuclear translocation [36,37]. PARP-1 can also interact with members of the NF-κB family favoring the formation of the transcription complex, independently of its enzymatic activity [7]. Other studies demonstrated that PARylation sustains p65 NF-κB activation and nuclear retention by reducing its interaction with nuclear exporting proteins [37,38]. Noteworthy, PARP-1 interacts with the histone acetyltransferases p300, a transcriptional co-activator required for NF-κB-dependent gene transcription [39]. Furthermore, acetylation of PARP-1 by p300 is required for full NF-κB-dependent transcriptional activity [17]. The relevance of PARP-1 in NF-κB activation and inflammation is clearly exemplified by the resistance of PARP-1KO mice to LPS-induced septic shock [40].

PARP-1 is a leading factor in oxidative stress-induced inflammation, being activated by and further fostering generation of reactive oxygen/nitrogen species (ROS/RNS). PARP-1 regulates the production of several inflammatory molecules including transcription factors, cytokines, chemokines, cyclooxygenase-2, iNOS. Inflammation generates additional ROS establishing a circuit that further sustains this response. ROS can induce mutations, epigenetic changes, and post-translational modifications resulting in alterations of expression and/or functions of several proteins [41,42]. Indeed, during chronic inflammation there is an accumulation of DNA damages which activates repair mechanisms [43]. PARP-1 plays a pivotal role interconnecting and sustaining reciprocal amplification of DNA damage, inflammation and cell necrosis leading to degenerative processes that can induce further activation of PARP-1. Generation of ROS and RNS is involved in several pathogenic processes sustained by PARP-1, including ischemia reperfusion injury, stroke, myocardial infarction, and neurodegenerative disorders [44,45,46]. 

Massive PARP activation results in NAD^+^ and ATP depauperation, organ dysfunctions [47], and induction of necrosis [48]. PARP-1 can also induce apoptosis by stimulating the release of the apoptosis-inducing factor (AIF) by mitochondria, through caspase-dependent and -independent pathways [49,50,51]. Noteworthy, PARPis can shift necrosis to apoptosis, at least under certain conditions [52].

PARs released by cleavage of PARylated proteins have been considered to act intracellularly, being recognized by specific motifs present on other proteins. More recently, PARs were shown to play a role also in cell-to-cell communication. Indeed extracellularly released PARs can stimulate mouse and human macrophages inducing cytokine and chemokine production [53]. PARs, released by damaged cells, could therefore act as a damaging associated molecular pattern (DAMP). DAMPs, or alarmins, are perceived as signals of danger by immune cells resulting in inflammatory response, release of chemotactic factors, leukocyte recruitment, and activation [54]. PAR also activates a form of cell death named parthanatos, as it occurs in neurons [55]. Parthanatos, as other forms of necrosis/necroptosis, results in the release of intracellular components, among which several DAMPs. Upon PARylation, HMGB1, an alarmin passively released during necrosis, is actively translocated from the nucleus to the cytoplasm and then released [56]. Noteworthy, PARylated HMGB1 also inhibits efferocytosis, reducing the clearance of apoptotic cells in damaged tissues, and therefore, sustaining inflammation [57]. Released HMGB1 is recognized by RAGE (Receptor for Advanced Glycation Endproducts) and TLR4 (Toll-Like Receptor 4). Moreover, free PARs are recognized by TLR4 (and TLR2), confirming convergence of these pathways in danger signaling [53]. As TLR4 engagement by HMGB1 or by LPS sustains HMGB1 PARylation and thus its release, this circuit represents one of the amplification loops hold up by PARP-1 in inflammation. Noteworthy, S. pyogens releases a NAD^+^ glycohydrolase into the host cell that reduces PARP-1 activation and accumulation of PAR and interferes with inflammatory cytokine signaling and HMGB1 release [58]. PARP-1 and HMGB1 are also targeted by other pathogens such as gammaherpesviruses [59] and Chlamydia trachomatis [60] as a strategy to limit inflammation and evade immune response.

**Figure 2:**
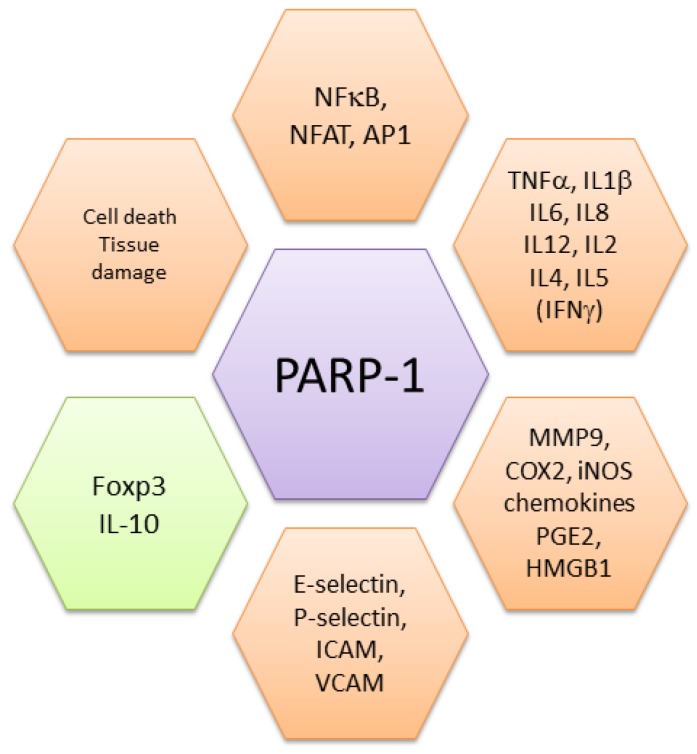
PARP-1 is ubiquitously expressed and plays a central role in inflammation (orange hexagons). By activating NF-κB through several mechanisms (and also NFAT and AP1), PARP-1 induces the production of inflammatory (TNFα, IL1β and others) and effector T cell cytokines (IL4, IL5). Inflammatory mediators activated by PARP-1 include metalloproteinases (MMP9), inducible nitric-oxide synthase (iNOS), several chemokines, prostaglandins (PGE2) and alarmins (HMGB1). It also favors cell recruitment through upregulation of adhesion molecules, including selectins and cell adhesion molecules (ICAM, Intercellular Cell Adhesion Molecule; VCAM, Vascular Cell Adhesion Molecule). When over-activated, PARP-1 also induces cell death and tissue damage, further fueling the inflammatory process. On the other side, PARP-1 inhibits the expression of Foxp3, a transcription factor required for regulatory T cell differentiation and function, and of IL10, an inhibitory cytokine (green hexagon).

Altogether, these findings demonstrate that by up-regulating danger signals, PARP-1 and PARylation are key players in several aspects of inflammation (Figure 2). Whereas at a physiological level, their effects are required to create the conditions for initiating and sustaining the (innate) immune response, their prolonged action is at the basis of several pathogenic processes (see below).

## 4. Pathogenic Role of PARP-1 in Cancer

### 4.1. High Activity of PARP-1 in Tumor Development and Progression

In considering the multifaceted role of PARP-1, it is worth mentioning that many reports showed increased levels of PARP-1 or, more generally, pointed to PARP-1 involvement in carcinogenesis, as for instance is the case for primary prostatic cancer [61]. PARP-1 has also been involved in prostate cancer progression, as PARP-1 expression in the nuclear matrix increases with tumor invasiveness [62]. Finally, both enzymatic activity and transcriptional regulatory functions of PARP-1 were reported to be elevated as a function of prostate cancer progression, independently of DNA double strand breaks, but through enhancement of E2F1–mediated induction of DNA repair factors involved in HR [63]. Elevated PARP-1 mRNA and protein are associated with poor prognosis in gastric cancer [64,65]; PARP-1 mRNA is elevated in colon carcinoma when compared to adenoma [66]; PARP-1 gene expression is associated with lymph node spread of malignant pleural mesothelioma [67]; and PARP-1 mRNA and protein are elevated in endometrial adenocarcinoma [68]. Both PARP-1 mRNA and protein are highly expressed in small cell lung cancer [69], but PARP-1 protein has been shown to associate with longer progression free survival (PFS) in limited-stage small cell lung cancer [70]. High PARP-1 protein resulted to be associated with shorter survival in soft tissue sarcomas [71] and an independent prognostic factor for decreased PFS and overall survival in high-grade serous ovarian carcinoma [72]. Furthermore, PARP-1 overexpression was associated with higher grade, estrogen receptor negativity, and triple negative (TNBC) as well as disease-free and overall survival in operable invasive breast cancer [73]. It is also associated with poor prognosis in oral squamous cell carcinoma [74]. Additionally, PARP-1 protein is higher in TNBC specimens than in non-TNBC breast cancers, and high PARP-1 expression is associated with worse PFS in TNBC [75]. On the whole, these studies indicate that elevated PARP-1 protein occurs in many tumor types suggesting an involvement in the oncogenic process, and may have prognostic value.

### 4.2. Oncogenesis in PARP-1KO Mouse Models

As widely discussed, PARP-1 plays a large role in genome maintenance (DNA repair, chromatin remodeling, transcription factor regulation), and also contributes to the propagation of the inflammatory phenotype. Clinically, the focus of PARP-1 is as a target for the treatment of familial cancers, such as BRCA1/2 deficient breast and ovarian tumors [76] but the possible therapeutic applications of PARPis extend far beyond cancer therapy to other types of stress-related diseases, and virtually any disease caused by acute or chronic inflammation (see above). Therefore, given the complex and multifaceted role played by PARP-1, clarifying the effect of PARP-1 abrogation in carcinogenesis is crucial in view on the possible opposite outcomes of its inhibition. In fact, while DNA repair failure consequent to PARP-1 abrogation might increase mutations frequency promoting tumorigenesis, inhibition of inflammatory pathway may be protective as inflammation is now recognized as a cancer hallmark [77].

A number of PARP-1KO mouse models were generated by different groups [78,79]. Genetically engineered mice have proven crucial to study the integrated functions of PARP-1 in multiple organs. Despite its important role in cellular response to genotoxic stress, PARP-1 is not required for viability, and mice lacking functional PARP-1 develop normally and are not predisposed to early-onset tumors. PARP-1KO mice, however, show hypersensitivity to ionizing radiation and alkylating agents, and PARP-1KO cells exhibit chromosomal instability, shown by increased frequency of spontaneous sister chromatid exchange and DNA damage-induced micronucleus formation [79]. In line with these findings, and with its role in DNA repair, PARP-1 deficient mice exhibit hypersensitivity to DNA damaging agents, with increased genomic instability and carcinogenesis at different sites [80,81]. Although PARP-1KO mice do not often develop spontaneous tumors, in a DNA repair deficient background, they develop tumor at high frequency. For instance, in p53 heterozygous and homozygous background, PARP-1KO mice developed a variety of tumors, such as mammary gland, lung cancer as well as brain tumors, including medulloblastoma (MB), a cerebellar pediatric tumor [82]. This indicated a synergistic functional interaction between PARP protein and p53 in tumor suppression through the role of PARP-1 in the DNA damage response and genome integrity surveillance. Functional interaction of PARP-1 with different NHEJ proteins has been described, suggesting a role of PARP-1 in NHEJ. For example, in the Ku80^-/−^ background, a high frequency of liver cancer was observed [83]. Moreover, PARP-1^−/−^/SCID double mutants, carrying a mutation in the gene encoding the catalytic subunit of the DNA-PKCs, show a marked increase in the frequency of T-cell lymphoma [84]. Interestingly, we showed that when PARP-1KO mice were crossed with Ptch1^+/−^ mice, a cancer prone mouse model in which induction of DNA damage accelerates development of cerebellum and skin malignancies, double mutants further accelerated medulloblastoma (MB) and basal cell carcinoma development following irradiation, providing evidence that PARP-1 function suppresses Ptch1-associated tumors arising in response to environmental stress [85]. By using the Ptch1^+/−^ mouse model, our group also investigated the effects of combined loss of the HR factor *Rad54* and *Parp-1*, highlighting novel synthetic lethal interactions during development, characterized by marked growth delay culminating in a perinatal lethal phenotype. This was associated with the ac cumulation of p53 and p21, causing enhanced senescence in MEFs and several tissues from compound mutants. Finally, the p53-dependent apoptotic/senescent phenotype was also tumor protective, suppressing radiation-induced MB tumorigenesis in *Rad54^−/−^*/Ptch1^+/−^ mutants with only one copy of *Parp-1* [86]. In addition, PARP-1KO animals, treated repetitively with the N-nitroso compounds-related carcinogen azoxymethane (AOM) were shown to develop an elevated number of colonic and liver tumors, implicating PARP-1 in the defense against N-nitroso compounds-induced colorectal and hepatic carcinogenesis [87].

In contrast, other recent reports raised the possibility of PARP-1 as a pro-tumorigenic factor, as many tumor types expressed high levels of PARP-1 [88], and its over-expression often correlates with disease progression [73]. Furthermore, loss of PARP-1 has been reported to decrease tumor development in vivo in several mouse models. Mice deficient in PARP showed decreased susceptibility to skin cancer through decreased NF-κB [35]. A novel role for PARP-1 in pancreatic cancer has been proposed based on the observation that PARP-1 depletion in the Ela-myc mice, a pancreatic cancer progression model, decreased the frequency of early stages of the disease and might be therefore be beneficial in preventing its development and progression [89]. In addition, PARP-1KO mice subjected to chemically-induced (AOM/dextran sodium sulfate) colorectal carcinogenesis were protected from tumor development compared to WT mice that carried significantly more tumors with a more aggressive phenotype characterized by upregulation of cyclin D1 and STAT3 [90]. Furthermore, in the same study, when PARP-1KO mice were crossed to O^6^-methylguanine-DNA methyltransferase (MGMT) null mice hypersensitive to AOM, double mutants (*Parp-1^−/−^/Mgmt^−/−^*) developed more but smaller tumors compared to the single mutants (*Mgmt^−/−^*), suggesting that PARP-1 has a double faced role in colorectal carcinogenesis by suppressing tumor initiation dependent on MGMT following DNA alkylation but promoting tumor progression. Of note, the protective effect conferred by the lack of PARP-1 was causally linked to the antinflammatory side of PARP-1 inactivation as in pancreatic carcinogenesis the blockade of tumor progression was associated with impaired macrophage recruitment, in colorectal tumors with downregulation of the IL6-STAT3-cyclin D1 axis, and in skin tumors with reduced NF-κB signaling. Altogether, these data show that PARP-1 genetic KO was not conclusive in respect to the inhibitory/promoting effect on cancer development, probably depending on the multifaceted role of PARP-1 in DNA repair and inflammation with possible opposite effects on tumor initiation and tumor promotion.

## 5. Pathogenic Role in Non-Cancer Diseases

Due to its role in oxidative stress and inflammatory responses, PARP-1 is also involved in several diseases other than cancer. As discussed above, PARP-1 over activation leads to further amplification of inflammation, cell death, and tissue degeneration. PARP-1 is involved in gene expression and activation of innate (neutrophils, macrophages, dendritic cells, microglia) and adaptive (T and B lymphocytes) immune cells [2,91,92]. PARP-1 pro-inflammatory action plays a relevant role also in non-immune cells, including endothelial cells, fibroblasts, and astrocytes largely contributing to the inflammatory response in virtually all tissues [93,94]. It is therefore not surprising that PARP-1 is involved in diseases sharing inflammatory/immune-mediated pathways, such as arthritis, diabetes, neurodegenerative disorders, colitis, and others, some of which are also linked to cancer development [32,44,45,46,95].

The role of PARP-1 in auto-reactive immune responses was shown for the first time in experimental models of rheumatoid arthritis (RA), a Th1 cell driven inflammatory autoimmune disease. In humans, an association between PARP-1 gene polymorphisms and RA was shown even if other studies reached contrasting conclusions [96,97]. A more recent study showed that a human RA-risk-associated non-coding polymorphism in the chemokine receptor CCR6 is a causal variant through which PARP-1 regulates CCR6 expression [98]. In gastric chronic inflammation induced by *Helicobacter*-specific T cell responses, PARP-1 sustains Th1 cell differentiation and the consequent T cell-driven immunopathology. By dampening this process, PARP inhibition not only prevents the formation of gastric precancerous lesions, but it also efficiently reverses pre-existing lesions, confirming its double role in inflammation and cancer [99].

PARP-1 is also involved in acute and chronic inflammatory bowel disease (IBD), as in the inflamed colon it induces cell death, activates NF-κB and AP-1, and sustains inflammatory cytokine production [100,101]. The role of PARP in intestinal inflammation is also due to its effects on gut microbioma composition as shown in PARP-1 deficient mice or through its enzymatic inhibition [102,103]. We found that PARP-1 inhibits the differentiation of Foxp3^+^ regulatory T cells (Treg), which are devoted to control the amplitude and duration of inflammatory/immune responses [104]. PARP-1KO mice express Foxp3 at higher levels and generate more inducible regulatory T cells than wild type cells [104], keeping under control the inflammatory responses induced by dextran sodium sulfate [102]. PARP-1-sustained chronic inflammation is associated with colorectal cancer progression both in mice and humans [90,105]. Thus, in this context, PARP-1 plays a double role: by fueling inflammation, it promotes colitis and possible cancer progression; on the other hand, it contributes to genome stability, and therefore, cancer prevention (see also above).

PARP-1 also sustains Th2 type inflammation, in particular in allergic responses and asthma. Genetic ablation of PARP-1 compromises the ability of naïve CD4 cells to differentiate into Th2 type inflammatory effector cells, reducing GATA-3 expression, and IL4 and IL5 production [106]. In murine models, upon allergen challenge, expression and activity of PARP-1 increase [107,108]. PARP-1 sustains ROS/RNS species production in alveolar macrophages, NF-κB activation, and expression of inflammatory mediators leading to chronic lung inflammation. In sensitized mice genetic ablation of PARP-1 or its enzymatic inhibition reduces inflammation and neutrophil infiltration, ameliorating allergic airway reactions, dyspnea and asthma-associated remodeling [109,110]. In humans, a PARP-1 gene polymorphism (Val762Ala) was reported to be associated with a decreased risk of asthma [111], while PARP-1 activation is increased in PBMC and lung tissues from asthmatic patients [112].

PARP-1 plays a relevant role in the oxidative/nitrosative stress following infarction-reperfusion and in septic shock. Reperfusion of patients that underwent coronary intervention leads to immediate PARP activation in peripheral blood mononuclear cells [113]. In patients that died, histological analyses showed that the presence of PARs correlates with inflammatory infiltration and the degree of myocardial dysfunction [114]. Upon reperfusion, PARP-1 activation also occurs in glial cells and neurons from patients that underwent ischemia due to cardiac arrest [115]. These findings underline a double detrimental effect of intense PARP-1 activation: cell death due to NAD^+^ depauperation and, in surviving cells, activation of a pro-inflammatory program leading to cell infiltration and further loss of function. PARP-1 is also involved in neurodegenerative diseases characterized by cytotoxic protein aggregates, including Parkinson’s disease (PD) and Alzheimer’s diseases (AD). In both diseases, PARP-1 is activated by and leads to the generation of ROS/RNS, DNA damage, cell death, and inflammation [116]. In PD human brain specimens, a significant increase of PARP-1 protein levels was revealed in dopaminergic neurons of the substantia nigra, associated with NF-κB nuclear translocation [117]. In mouse models, aggregates of α-synuclein activate PARP-1. Consequent PAR generation accelerates α-synuclein misfolding and aggregation, resulting in the induction of parthanatos and loss of neurons. Inhibition of PARP activity or *Parp-1* gene deletion breaks this circuit preventing neuron-to-neuron transmission of pathologic α-synuclein and neurotoxicity. The relevance to human disease is sustained by the high levels of PAR in brains and cerebrospinal fluid from PD patients [118]. Enhanced PARP-1 activity and PAR accumulation were also observed in brains from AD patients [119]. In cell cultures, β-amyloid activates PARP-1 in astrocytes leading to indirect death of hippocampal neurons. PARP enzymatic inhibition prevents cell death and further neuronal damage [120]. Noteworthy, specific PARP-1 haplotypes were reported to be associated with the development of AD [121].

PARP-1 activity was also associated with multiple sclerosis (MS), another inflammatory-neurodegenerative disease. MS patients show higher PARP-1 activity in monocytes while its enzymatic inhibition in cell cultures reduces neuroinflammation-associated pathways [122,123]. A clear role for PARP-1 in neuroinflammation was demonstrated in the experimental autoimmune encephalomyelitis (EAE) model in mouse and confirmed in non-human primates. In these settings, in addition to its effects on ROS, PARP-1 also regulates dendritic cell migration and T cell activation [95,124,125]. Noteworthy, oxidized derivatives of cholesterol are present at high levels in cerebrospinal fluid from multiple sclerosis patients and from mice with EAE. These cholesterol byproducts activate microglia, macrophages, and also astrocytes, leading to inflammation, leukocytes recruitment and neuronal damage, through mechanisms involving PARP-1 [122,126]. Inhibition of PARP reduced the expression of iNOS and CCL2, but did not affect IFNγ and IL-17 production, both cytokines being considered relevant to the encephalitogenic process [122,127]. No significant differences in IL-17 production were also observed in an imiquimod-induced model of psoriasis, a finding deserving further studies to clarify the role of PARP-1 in Th17-cell driven inflammation. Curiously, PARP-1 depletion enhanced the severity of psoriasis-associated inflammation [128]. Moreover, ex vivo stimulation of purified naïve CD4 cells from PARP-1KO mice generates Th17 cells with a frequency similar to wild type cells [104]. Activation of iNOS by PARP-1 and neuronal damage lead to an increase in oxidative stress, that will generate further byproducts and activation of PARP-1, establishing a vicious circuit. PARP-1 therefore plays a composite role in inflammation by sustaining ROS generation, activation of transcription factors, expression of several inflammatory mediators, recruiting inflammatory cells, activating lymphocytes, and limiting negative regulation of responses (Figure 2).

## 6. Therapeutic Implications of PARP Inhibitors

The involvement of PARP-1 in DNA damage detection and repair mechanisms has stimulated an intense research aimed at developing pharmacological inhibitors, during more than 50 years of research since the initial discovery of this important class of enzymes. PARPis are small molecule NAD^+^ mimetics differing in specificity and potency that bind to the NAD^+^ site in the catalytic domain of PARP-1, preventing PARylation through catalytic inhibition. Structurally, all the PARPis that entered clinical trials contain nicotinamide-mimic motifs that compete with the nicotinamide pocket of NAD^+^. PARP-1 self-PARylates its auto-modification domain to release itself from DNA, a process that when inhibited results in PARP-trapping [12]. Although the precise mechanisms that explain PARP-1 trapping are still unclear, two have been proposed: (i) PARPis either prevent the release of PARP-1 from DNA by inhibiting autoPARylation [129] or (ii) PARPis binding to the catalytic site causes allosteric changes in the PARP-1 structure enhancing DNA avidity [130]. Whatever the origin, trapped PARP-1-DNA complexes were more cytotoxic than unrepaired lesions caused by PARP-1 inactivation [131] and stalled the progression of replication fork resulting in their collapse into lethal DNA damage. Over the years, nicotinamide and 3-aminobenzamide, representing the first generation of inhibitors active at millimolar concentrations, were replaced by 2-nitro-6[5H] phenanthridinone, 1,5-dihydroisoquinoline, and others, active at mid-micromolar concentrations. The present third generation includes inhibitors effective at low micromolar/high nanomolar concentrations, several of which proceeded to clinical development or were already approved by regulatory agencies [132].

Whereas the NAD^+^-dependent route of PARP-1 activation has been exhaustively exploited for designing new inhibitors, in the attempt of finding less cytotoxic inhibitors, more selective toward PARP-1, non- NAD^+^ PARP-1 inhibitors, which work by different mechanisms of action of NAD^+^ analogous, started to be developed and are currently under active experimentation [133,134]. 

### 6.1. Therapy of Cancer

The recent approvals of PARPis olaparib (Lynparza) and rucaparib (Rubraca) for treating BRCA-mutated ovarian cancer [135] and niraparib for the treatment of recurrent ovarian cancer with or without BRCA mutation [136] are important benchmarks. Olaparib was the first PARPi approved by the Food and Drug Adiministration in December 2014 for use as monotherapy in patients with germline BRCA mutated (gBRACm) advanced ovarian cancer [137]. Since then, the field of PARPi monotherapy has rapidly advanced, with additional FDA approvals of niraparib (March 2017) and rucaparib (April 2018) for maintenance treatment of BRCA-mutated ovarian cancer. In 2018, two PARPis were approved for gBRCAm metastatic breast cancer, olaparib, and talazoparib [138]. There are now more than 150 completed, running, or planned registered trials for the PARPis niraparib, olaparib, rucaparib, talazoparib, and veliparib (www.clinicaltrials.gov). The inhibition of PARP-1 is being exploited for the treatment of various cancers, which include DNA repair-deficient ovarian, breast, and prostate cancers. PARPi clinical trials are now expanding to include various solid tumors such as pancreatic, biliary, urothelial, NSCLC, liver, colorectal, oesophageal, gastric, uterine, carcinosarcoma, brain metastasis Ewing’s sarcoma, and others [134] (www.clinicaltrial.gov). The majority of these trials are monotherapy studies in patients with tumors harboring DNA repair defects while the remaining trials are combinations with chemotherapies, including platinums, taxanes, ATM (ataxia telangiectasia, mutated) inhibitors, ATR (ATM and RAD3-related) inhibitors, Wee1 inhibitors, and PI3K inhibitors, as well as with immune-oncology therapies. Of particular interest is the combination of PARPis with the immune checkpoint inhibitors. PARP inhibition promotes differentiation of naïve T cells to Foxp3^+^ regulatory T cells [104], which suppress immune responses, and upregulates in tumor cells the expression of PD-L1 [139,140], which by engaging PD1 dampens anti-tumor T cell responses favoring tumor immune-evasion. Combined therapy using anti-PD-L1/anti-PD-1 blocking antibodies and PARPis showed synergic effects in mouse models [140] and promising antitumor activity in clinical trials [141].

Although PARP inhibition is a promising therapeutic approach for *BRCA*-mutated cancers, in some cases, PARPi resistance can emerge, through several and often poorly understood mechanisms. Inactivation of p53-binding protein 1, genetic reversion of *BRCA1* or *BRCA2* mutations, elevated levels of components of the HRR pathway such as RAD51, as well as point mutations in PARP-1, and depletion of PARG, the major enzyme involved in the catabolism of PARP, are among the proposed mechanisms of resistance [138].

The most concerning potential adverse reactions associated with PARP inhibition are myelodysplastic syndrome and acute myeloid leukemia (MDS/AML), especially in patients harboring a germline *BRCA* mutation [142]. The U.S. Package Insert for Lynparza (olaparib) contains the following warning for the development of MDS/AML: MDS/AML have been confirmed in six out of 298 (2%) patients enrolled in a single arm trial of Lynparza monotherapy, in patients with deleterious or suspected deleterious germline *BRCA-*mutated advanced cancers [LYNPARZA™(olaparib): http://www.accessdata.fda.gov/scripts/cder/drugsatfda/]. Bone marrow toxicity, including cytopenias, has also been reported [143].

One of the challenges is how to recognize patients who will benefit from the use of PARPis from those that will suffer from adverse severe reactions, including secondary malignancy. The current PARPis lack of selectivity between PARP-1 and PARP-2, and this could increase cellular toxicity as demonstrated by the result of an in vivo study showing embryonic lethal phenotype in double PARP-1 and PARP-2 knockout mice [144]. To obtain greater selectivity for PARP-1, novel next generation specific PARP-1i are being developed that target activation mechanisms unique to PARP-1 enzyme. This consist in either developing more specific NAD^+^ such as PARPis by exclusively targeting amino acids of the adenosine-binding site of PARP-1 along with residues of nicotinamide binding site, as well as non- NAD^+^ PARP-1is which work by different mechanisms of action of NAD^+^ analogous [134].

### 6.2. Therapy of Non-Cancer Diseases

As discussed above, preclinical studies demonstrated that PARP plays a role in almost all acute and chronic inflammatory/immune-mediated diseases, with different etiopathogenesis and involving different organs or being systemic. For many oxidative/inflammatory disorders an involvement of PARP-1 was also demonstrated in humans as discussed above. In animal models, studies aimed at controlling rheumatoid arthritis and cell damage by ischemia/reperfusion through inhibition of PARP-1 activity began as early as in the 1990s, using first (such as nicotinamide) and second generation (PJ34, DPQ) pharmacological inhibitors [145,146]. Interestingly, in inflammatory bowel disease, beneficial effects were obtained with both PARP and PARG inhibitors, the latter compromising PAR degradation and thus sustaining PARP-1 auto-inhibition [101,147]. The effectiveness of clinically relevant inhibitors in many in vitro and in vivo models of inflammatory diseases has confirmed and further corroborated the hypothesis of beneficial effects of PARP inhibition therapy in humans. Studies include results relevant to stroke, neurodegeneration, neuroinflammation and blood brain barrier function [148,149], sepsis [150], liver diseases [151,152], and asthma [112]. Protection of human neuronal cells by in vitro oxidative stress or NMDA was shown with rucaparib, veliparib, talazoparib, and olaparib [153].

Protective effects of PARPis in inflammation-mediated acute and chronic diseases could derive from several mechanisms: reduction in NAD^+^ consumption and prevention of energetic failure and consequent cell death; reduced AIF release by mitochondria and prevention of parthanatos; drastic attenuation of oxidative stress responses; decreased activation of NF-κB and consequent reduction in inflammatory cytokine expression and release of DAMPs; reduced expression of adhesion molecules and inflammatory infiltrate; possible reduction in inflammation-/oxidative stress-induced genotoxicity [154,155]. When PARPis are used, all of these mechanisms can be targeted at the same moment with synergic effects, this consideration explaining why in non-oncological studies effective doses of PARPis are lower than doses used in cancer settings.

**Figure 3:**
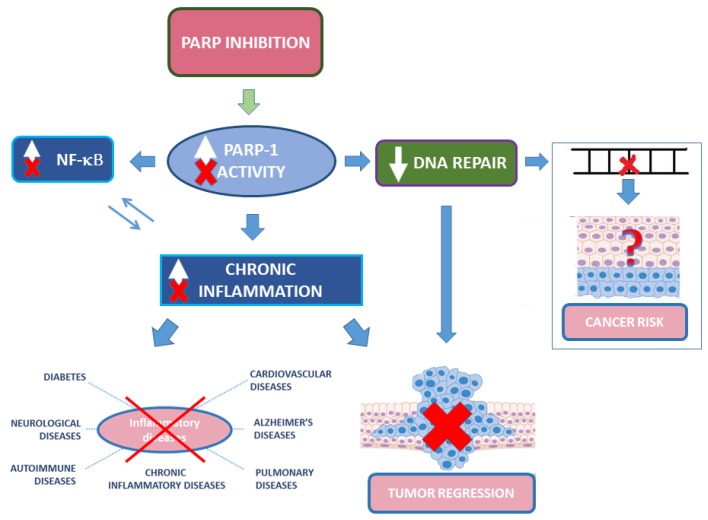
Schematic representation of the effects of PARP inhibition on cancer and inflammatory-related diseases. PARP inhibition reduces NF-κB activation and inflammation, with beneficial effects in inflammatory diseases and cancer. Yet, inhibition of DNA damage recognition and repair, although beneficial in cancer therapy, might increase cancer risk.

Although PARP-1 is not a proper DNA repair enzyme, its role in damage recognition and recruitment of repair enzymes raise concerns on the use of PARPis in non-oncological diseases [156]. Important issues are the potential side effects of long-term treatment and whether PARP inhibition may increase the risk of mutagenesis or oncogenesis (Figure 3). Indeed, PARPis lead to genotoxic effects in vitro. Yet, considerations on in vivo relevance of these studies and possible indirect protection from oxidative response-induced DNA damage make the picture more complex [157,158]. Although safety studies required for oncological drugs are “lighter” than for other diseases, clinically relevant PARPis passed safety studies and their potential side effects should be compared with currently available therapies for the considered diseases (for instance methotrexate in autoimmunity), none of which devoid of relevant side effects. Considering that for many inflammatory diseases there are no resolving therapies, the use of PARPis could be envisaged, provided an adequate risk/benefit analysis. Priority could be given to those non-oncological diseases that would require a short term PARP inhibition in acute phase(s), using more caution in long term treatments. An exhaustive and detailed analysis on the use of PARP inhibitors, unmet needs in non-oncological diseases, and the potential repurposing for the therapy of non-oncological diseases of PARPis was recently published [155].

## 7. Conclusions and Perspectives

PARP-1 is the best-studied PARP enzyme, which is also the most ubiquitous and abundant PARP protein. PARP-1 belongs to the DNA-dependent nuclear PARPs group whose catalytic activity is potently stimulated by DNA breaks. However, over the years, PARP-1 functions have been expanded with roles in DNA damage repair as well as transcription, chromatin structure, and metabolism. Thus, PARP-1 appears to be involved in both basal processes and response to cellular stresses with implications in multiple diseases, including cancer. Following FDA approval, PARPis have entered clinical trials for ovarian and breast carcinomas. However, side effects such as cytopenia, fatigue, and nausea, as well as more serious consequences consisting of secondary malignancies, such as myelodysplastic syndrome/acute myeloid leukemia (less 2%), have also been reported. The physiological functions of PARP-1 in vivo remain enigmatic, and despite remarkable progresses, even a simple approach as targeting PARP-1 through genetic KO was not conclusive in respect to the inhibition or promotion of cancer development. This is likely to be dependent on the multifaceted role of PARP-1 in DNA repair and inflammation that might have opposite effects in tumor initiation and tumor promotion. It has been proposed that PARPis developed as cancer treatments could also be used in the therapy of inflammatory diseases, including neurological disorders, as inhibition of ADP-ribosylation activity mitigates neurodegeneration in several animal disease models. However, whether long-term PARP inhibition would have detrimental effects on the normal brain function, for instance, remains an open question. Further insights into the function of PARP-1 in homeostatic conditions, which might better clarify the cause and effects of its dysregulation in pathological states, as well as more follow-up data on secondary malignancies in PARPis-treated cancer patients are needed, before widening the eligibilities of PARPis to inflammatory diseases.
